# Neural differentiation in perspective: mitochondria as early programmers

**DOI:** 10.3389/fnins.2024.1529855

**Published:** 2025-01-08

**Authors:** Ramin M. Farahani

**Affiliations:** ^1^IDR/Research and Education Network, Westmead, NSW, Australia; ^2^Faculty of Medicine and Health, School of Medical Sciences, The University of Sydney, Sydney, NSW, Australia

**Keywords:** mitochondria, neural differentiation, electron transport chain, redox (bio) chemistry, thermal signal

## Abstract

Neural differentiation during development of the nervous system has been extensively studied for decades. These efforts have culminated in the generation of a detailed map of developmental events that appear to be associated with emergence of committed cells in the nervous system. In this review the landscape of neural differentiation is revisited by focusing on abiotic signals that play a role in induction of neural differentiation. Evidence is presented regarding a chimeric landscape whereby abiotic signals generated by mitochondria orchestrate early events during neural differentiation. This early stage, characterised by mitochondrial hyperactivity, in turn triggers a late stage of differentiation by reprogramming the activity of biotic signals.

## Introduction

**Figure 1:**
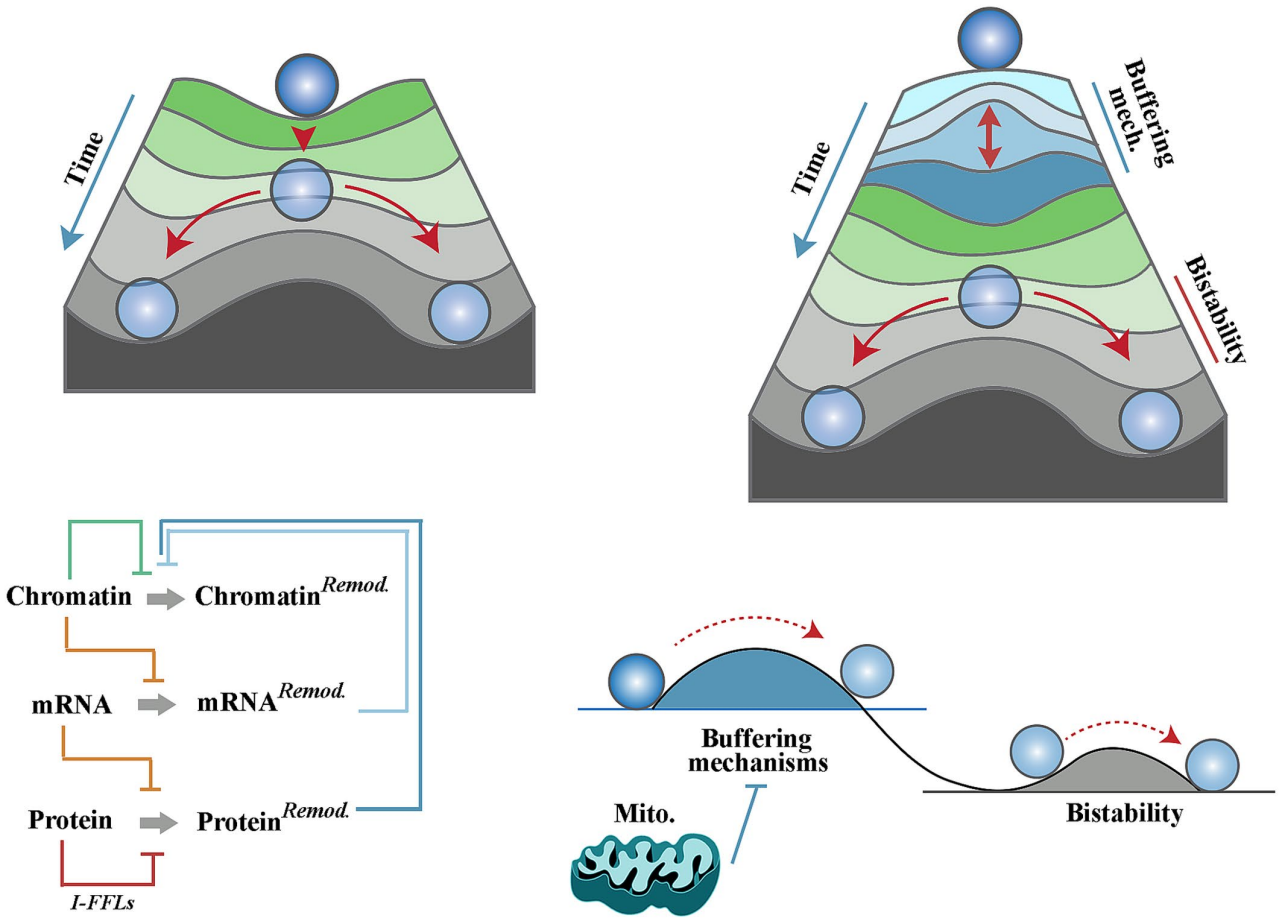
Mitochondria and a chimeric landscape of neural differentiation. Waddington’s landscape of cellular differentiation (top left) as opposed to the modified Waddington’s model (top right) whereby multiple homeostatic buffering mechanisms give rise to a differentiation hurdle that must be overcome prior to induction of differentiation. These mechanisms (outlined in the bottom left diagram and explained in the text) enforce network resilience of biotic signalling pathways and hence prevent uncontrolled differentiation of neural progenitor cells. In the modified model, mitochondria suppress the homeostatic buffering mechanisms and facilitate induction of differentiation (bottom right).

Cell differentiation, defined as an assumption of a specialised cellular task, is a hallmark of development. Performance of a specialised task is typically driven by reprogramming the proteomic landscape of a multipotent cell (Chaerkady et al., 2009; Samuels et al., 2024). This in turn requires a rewiring of the existing transcriptomic landscape (Kosak and Groudine, 2004; Wu et al., 2010) and a reciprocal modification of genomic/epigenomic profiles to consolidate the emerging transcriptome. Entanglement of these landscapes, however, poses a hurdle to cellular differentiation. The barrier stems from the network resilience (Wagner, 2008) of neural progenitor cells due to compensatory feedback between genomic, transcriptomic and metabolomic landscapes. Built into signalling network topologies of progenitor cells, feedback loops prevent significant divergence from a state of equilibrium. For example, translational buffering is a mechanism that opposes the impact of alterations in mRNA levels on the proteome (Kusnadi et al., 2022). Aside from translational buffering, the impact of alterations of an mRNA level can be offset by adaptive upregulation of compensatory mRNAs that are functionally redundant with an affected mRNA (Sztal and Stainier, 2020). Finally, the existence of incoherent feed-forward loops (i.e., antagonistic elements) in signalling pathways enforces the equilibrium state by blocking downstream communication of isolated biological signals that are not authenticated by input from other converging pathways (Vujovic et al., 2023). The complexity of buffering mechanisms that are built into signalling pathways are beyond the limited examples provided herein and readers are referred to a review by Liu *et al*. for an in-depth discussion of the topic (Liu et al., 2022). A corollary of network resilience of progenitor cells is that a cell can only embark upon differentiation once it approaches a “tipping point” (Rezaei-Lotfi et al., 2021) characterised by interruption of the majority of buffering mechanisms discussed above (Figure 1). This argument, however, gives rise to a dilemma; how could biological differentiation-inducing signals drive a cell towards a tipping point of cessation of the buffering mechanisms while such signals are typically suppressed by the very same mechanisms? Biological differentiation-inducing signals remain elusive, and differentiation is typically analysed by triggering withdrawal of biological signals in well-defined *in vitro* models of differentiation. Here, the aim is to define basic tenets of a chimeric differentiation model whereby early events during neural differentiation are triggered by abiotic (i.e., biophysical) differentiation-inducing signals of mitochondrial origin. It is this early stage that primes the cell for differentiation by reprogramming the activity of biotic signals and thus driving progenitor cells towards the tipping point of differentiation (Figure 1).

## Biotic versus abiotic signals in neural differentiation

Metazoan biology has been dominated by analysis of biotic signals, whereas biotic and abiotic signals have been equally focused upon and studied in plant biology (Ku et al., 2018; Peck and Mittler, 2020). The dichotomy in part reflects uncoupling of animals from abiotic signals/stressors owing to metazoan-specific adaptive mechanisms such as locomotion, as an avoidance mechanism, as well as an effective circulatory system with thermal and redox buffering capacities. However, it can be argued that most of the adaptive mechanism are either absent or rather ineffective in early stages of development. A focus on emergence of a functional vascular system within developing brain clearly demonstrates this point. In a developing mouse brain, primitive vasculature originating from yolk sac infiltrates the neural tube at embryonic (E) day 8.5 (Ozsoy et al., 2021). However, a functional circulation is not established until E10 (McGrath et al., 2003). The primitive vasculature originating from yolk sac is not only devoid of an effective redox buffering (Kuhn et al., 2017) capacity, but also contributes to generation of abiotic redox signals by supplying heme to mitochondria of neuroepithelial cells and thereby energising the electron transport chain (Ozsoy et al., 2021). Blood vessels also provide a thermoregulatory capacity by dissipating generated heat. It is estimated that resting skin blood flow effectively dissipates the metabolic heat at a rate of 80 to 90 kcal/h (Charkoudian, 2003). Notably, in early stages of development and prior to establishment of a functional vasculature, zebrafish embryos generate heat at an approximate rate of 30 nW/cell (Rodenfels et al., 2019) (equivalent to a spike of ≈4.8 K/s in a cell exposed to such a thermal flux). Such thermal flux in the absence of vasculature is expected to elevate intracellular temperature with implications for differentiation of progenitor cells. Thermal energy functions as an abiotic signal that accelerates differentiation dynamics (Chuma et al., 2024). Diminished thermal signalling in mild hypothermia (35C), on the other hand, inhibits differentiation dynamics of stem cells (Belinsky and Antic, 2013). Hence, in the early stage of metazoan development and prior to establishment of functional homeostatic mechanisms, there appears to be a window of opportunity for abiotic signals to alter developmental dynamics.

## Thermal signalling and regulation of neural differentiation

Intracellular temperature oscillates within a defined range dependent on chemical reactions occurring within cycling cells (Rodenfels et al., 2019). Experimental measurement of heat exchange in zebrafish embryos at the 2-cell stage using microcalorimetry revealed that heat is generated at an approximate rate of 30 nW/cell (Rodenfels et al., 2019), equivalent to a temperature spike of ≈4.8 K in a period of one second in a thermodynamically closed system. Amongst generators of thermal energy, mitochondria appear to be particularly effective in raising the intracellular temperature. This is owing to the high rate of generation of thermal energy by these organelles. Near-complete conversion of mitochondrial output to heat by protonophores induces a temperature spike of ≈4.8 K/s (Rajagopal et al., 2019). However, a meaningful elevation of intracellular temperature is unlikely to last long. Given the estimated upper limit of a cell’s capacity for dissipation of Gibbs free energy (−3.7 kJ g^−1^ h^−1^) (Niebel et al., 2019), if heat is generated at a reported rate of 30 nW/cell (Rodenfels et al., 2019), a biologically meaningful temperature rise will only last seconds. While successive waves of mitochondrial activity could expand this hyperthermic window somewhat, such hyperthermia is unlikely to last longer than minutes (Lepock, 2004). In this light, it seems reasonable to ask how such transitory intracellular hyperthermia could contribute to neural differentiation (Nomura et al., 2022; Vujovic et al., 2022). Herein, this question is addressed by a focus on interactions of thermal energy and the Notch signalling pathway as a master regulator of stemness.

Notch signalling pathway is known to inhibit differentiation of neural progenitor cells (Imayoshi et al., 2010; Lampada and Taylor, 2023). Repression of neurogenesis may partially be attributed to trans-activation of the c-Myc gene (Palomero et al., 2006), a pro-anabolic regulator that enhances protein synthesis at G1 phase of cell cycle and amplifies proliferation rate of cycling cells (Zeller et al., 2006). Further, Notch-1 output appears to trigger expression of paradoxical (i.e., antagonistic) elements (Hart and Alon, 2013) that populate critical nodes in the mitogenic PI3k/Akt pathway (Vujovic et al., 2019). Convergence of the antagonistic outputs downstream to Notch signalling delays premature progression to S phase while protein synthesis continues. This mode of signalling results in emergence of bistability (Vujovic et al., 2019; Agrawal et al., 2009) (i.e., duality) whereby an apparent delay in progression towards a tipping point builds a dormant capacity by continued protein synthesis to facilitate cellular dynamics past the tipping point. In the context of differentiation, a bistable landscape manifests as induction of quiescence by high Notch signalling activity and assumption of a differentiated state upon downregulation of the signalling output (Ninov et al., 2012; Perdigoto et al., 2011). This line of reasoning places the spotlight on mechanisms that switch off the Notch pathway. While induction of a Notch^on^ state has been extensively studied (Bray, 2016), transition to a Notch^off^ state upon resolution of bistability remains understudied.

Notch, at a post-translation level, can be down-regulated by generic protein removal mechanisms of endocytosis (McGill et al., 2009), autophagy (Wu et al., 2016) and ubiquitination (Lai, 2002). By increasing membrane fluidity, hyperthermia enhances the rate of endocytosis (Chanaday and Kavalali, 2018). Likewise, autophagy appears to be positively affected by hyperthermia (McCormick et al., 2021). Ubiquitin, on the other, is a heat shock protein (Bond and Schlesinger, 1985) with temperature-dependent kinetics amplifying degradation of proteins at a higher heat flux (Parag et al., 1987). Despite this evidence, it remains to be investigated whether degradation of Notch or its intracellular domain increases in a heat-dependent manner. Direct evidence regarding thermal regulation of Notch signalling pathway can be found at the level of receptor/ligand interaction. In a hyperthermic condition, canonical signalling activity of the Notch pathway decreases leading to enhanced neural differentiation of chick neural progenitors. The attenuated signalling appears to be linked to an altered lipid composition of the plasma membrane which reduces cell–cell interactions and ligand-dependent Notch signalling activity (Nomura et al., 2022). Complementing the negative impact of hyperthermia on induction of the Notch^on^ state, heat flux promotes transitioning to a Notch^off^ state. To this end, the Ankyrin domain of Notch-1 functions as thermal receptor which harvests thermal energy and converts it to micromechanical oscillations (Vujovic et al., 2022). These vibrations lead to destabilization and dissociation of Notch1 transcriptional complex and transition from a Notch^on^ to a Notch^off^ state with resultant acceleration of neural differentiation (Vujovic et al., 2022). Collectively, emerging evidence suggests that thermal energy functions as an abiotic signal, with Notch signalling pathway evolved to interact with, interpret and integrate, the abiotic thermal signal to biotic signalling pathways. Aside from more specific interaction with Notch signalling pathway, thermal energy facilitates remodelling of the proteomic landscape of progenitor cells via autophagy and ubiquitination as outlined previously.

## Redox-mediated reprogramming of signalling pathways

Unlike thermal signalling, redox signalling has been extensively studied and readers are referred to other reviews covering this topic (Forman et al., 2014; Bindoli and Rigobello, 2013). Here, redox signalling is a reference to interaction of an electrophile (i.e., a electron acceptor) with a nucleophile (i.e., an electron donor) wherein the oxidised form of a nucleophile not only occupies a critical node within a signalling network but also whose activity is altered in an oxidised state. It appears that several signalling pathways have accommodated redox-sensitive proteins characterised by presence of conserved cysteine residues in active catalytic sites (Vujovic et al., 2023). Further, these redox-sensitive proteins are elements of incoherent feed-forward loops whereby an upstream stimulus generates two competing signals with opposing effects on a downstream target. In this configuration, a switch from the reduced form of a protein to the oxidised form alters the communication of upstream signals to downstream mediators. The. Network topology of PI3K/PTEN illustrates this notion. Upon activation, PI3K catalyses the conversion of PIP2 to PIP3 which prompts Akt signalling (Hemmings and Restuccia, 2012). Concurrent activation of PTEN by catalytic activity of protein phosphatase 2A (Nakahata et al., 2014) or by auto-dephosphorylation (Zhang et al., 2012) antagonises the function of PI3K by converting PIP3 to PIP2. In an oxidising milieu, PTEN becomes reversibly inactivated due to the formation of an intramolecular disulfide between the essential active Cys-124 residue and Cys-71 (Lee et al., 2002). This transient inactivation of PTEN is expected to facilitate downstream communication of signals.

In support of this notion, it has been demonstrated that platelet-derived growth factor (an upstream activator of PI3k/Akt) transiently increases the intracellular concentration of hydrogen peroxide and that neutralising this activity via antioxidants blunts the signalling activity of this growth factor. It is noteworthy that the redox-mediated reprogramming of signalling pathways is not limited to PI3k/Akt pathway. A detailed review of the concept can be found elsewhere (Vujovic et al., 2023).

In a similar manner to thermal signalling, a shift in the redox state is swift and short-lived. For example, the switch to the oxidised inactive form of PTEN takes minutes and the recovery of the reduced active form occurs within an hour (Zhang et al., 2020). The transient nature of this shift reflects the hyperactivity of defence mechanism that eliminate the oxidants. For example, superoxide dismutase converts O_2_^•−^ to O_2_ and H_2_O_2_ at a rate of 10^9^ M^−1^ s^−1^ (Forman and Fridovich, 1973). Given the short-lived nature of redox signals, one may ask if abiotic redox signals could plausibly contribute to neural differentiation. A scenario illustrating contribution of redox signals to neural differentiation occurs during vascularisation of neural tube at murine E8.5. During this period, a fraction of neuroepithelial cells of developing mouse brain transiently fuse with endothelial cells and internalise primitive erythroblasts while acquiring the heme content of these cells via transendocytosis (Ozsoy et al., 2021). Heme-mediated activation of mitochondria amplifies production of reactive oxygen species and accelerates differentiation of the cannibalistic cells (Ozsoy et al., 2021). From a mechanistic perspective, a central clue regarding the role of redox in neural differentiation is provided by focusing on redox-mediated rewiring of the PI3K/Akt pathway during neural differentiation. We know that abrogation of the PI3K/Akt signalling pathway effectively blocks neural differentiation of progenitor cells (Sanchez et al., 2004; Lopez-Carballo et al., 2002). Hence, a transient change in redox could be sufficient to reprogram the latter signalling pathways as discussed in a previous section and to facilitate neural differentiation of progenitor cells (Prozorovski et al., 2015). Interestingly, redox signalling complements the impact of thermal signalling. For example, the stability of Notch1 intracellular domain is regulated by activity of NAD^+^-dependent deacetylase Sirt1 (Guarani et al., 2011). While acetylated Notch1 intracellular domain exhibits a long half-life, deacetylation by Sirt1 destabilises the protein and facilitating elimination (Guarani et al., 2011). Therefore, conversion of NADH to NAD^+^ in an oxidising environment (Xiao and Loscalzo, 2020) and the resultant activation of Sirt1 enzymatic activity will destabilise Notch1 intracellular domain and thus complement temperature-mediated destabilisation of the RBPj-associated protein.

## Intracellular pH: an underrated player in differentiation?

The most convincing mechanistic evidence regarding the role of intracellular pH (with free protons as abiotic signals) in cell reprogramming was reported by McBrian et al. (2013). They demonstrated that a reduction of intracellular pH triggers global deacetylation of histones. Released acetate anions associate with protons and are exported out of the cell, a mechanism which buffers against further reduction of pH (McBrian et al., 2013). Therefore, a change of intracellular pH is linked to epigenetic reprogramming of histones. Given this evidence, it is hardly surprising that intracellular pH regulates intestinal stem cell lineage specification (Liu et al., 2023) and that blocking pH fluctuations impairs the differentiation program (Ulmschneider et al., 2016). Corroborating this notion, an adaptation that facilitates abiotic signalling activity of protons in neural progenitor cells is reduced buffering capacity of these cells compared to differentiated cells (Nordstrom et al., 2012).

## Mitochondria in the early differentiation landscape: generation of abiotic signals and attenuation of biotic signals

Mitochondrial electron transport chain components are major contributors to production of reactive oxygen species and a shift to an oxidising milieu. Electron leakage and production of reactive oxygen species occurs in the ubiquinone binding sites in complex I and complex III, glycerol 3-phosphate dehydrogenase, the flavin in complex I, the electron transferring flavoprotein:Q oxidoreductase of fatty acid beta oxidation and pyruvate and 2-oxoglutarate dehydrogenases (Brand, 2010). Aside from the steady-state (i.e., physiological) production of reactive oxygen species, mitochondria could be reprogrammed to accelerate transitioning to an oxidising state. One such occasion occurs during conversion of dihydroxyacetone phosphate to glycerol-3-phosphate to regenerate NAD^+^ in cells with a repressed electron transport (Liu et al., 2021). In this scenario, oxidation of glycerol-3-phosphate by mitochondrial glycerol-3-phosphate dehydrogenase in the mitochondrial intermembrane space leads to a significant leakage of electrons and the generation of reactive oxygen species, at levels comparable with the maximum rate of ROS generation reported for complex III when inhibited with antimycin A (Miwa and Brand, 2005). Mitochondria are also the major source of thermal energy within cells. Recent evidence shows that in normal physiological conditions mitochondrial temperature is close to 50°C when the electron transport chain is fully active (Chretien et al., 2018). In fact, maximal activity of various respiratory chain enzymes is observed at or slightly above 50°C (Chretien et al., 2018). The implications of this finding are explained in several current reviews (Lane, 2018; Macherel et al., 2021). An extension of the latter observation is that the thermal flux within a cell is expected to be affected by the fraction of mitochondria that are synchronously in state 3 (high respiratory chain activity). This in turn will depend on availability of electron transport chain substrates and on inter-mitochondrial communication of signals (Huang et al., 2013).

Regarding the source of intracellular free protons, intracellular acidification could occur consequent to mitochondrial metabolic activity or in response to the metabolic arrest of mitochondria. Upon mitochondrial metabolic arrest, the glycolytic production of lactate (pKa 3.86) would result in release of protons and a reduction of intracellular pH (Wu et al., 2007). On the other hand, CO_2_ generated in the tricarboxylic acid cycle is typically converted to carbonic acid, H_2_CO_3_, which then dissociates to HCO3^−^ and H^+^ (6 H^+^/glucose) (Mookerjee et al., 2015). Therefore, mitochondria with active electron transport chain contribute to the cytosolic proton pool in a manner similar to the generation of thermal energy and of reactive oxygen species.

**Figure 2:**
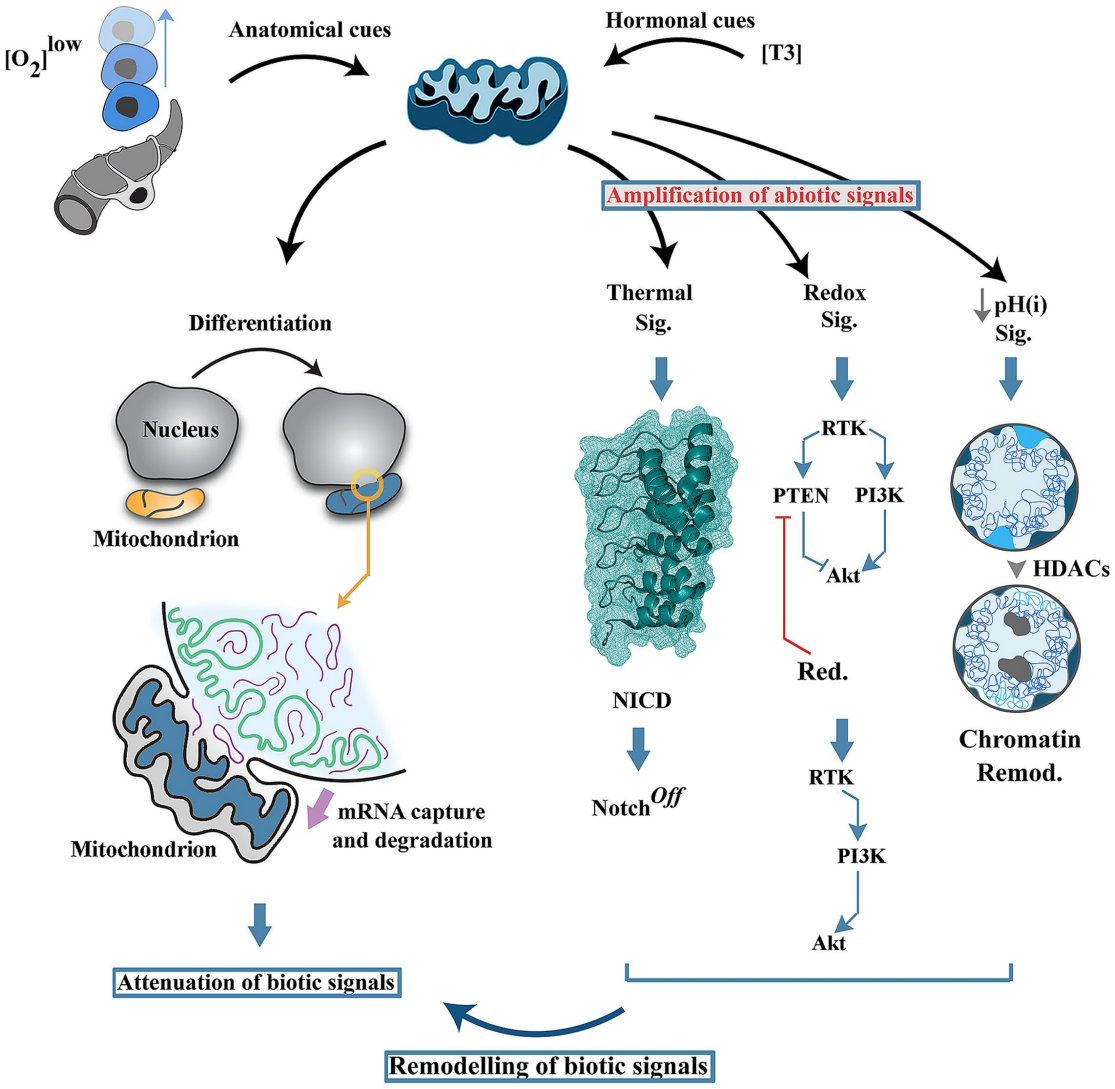
Mitochondria as early programmers of neural differentiation landscape. Induction of differentiation by local cues (e.g., hypoxia) or by endocrine mediators (e.g., T3) triggers transient fusion of mitochondria and nucleus leading to acquisition and degradation of nuclear-enocoded RNAs. In parallel, generation of abiotic signals by mitochondria (i.e., thermal flux, redox signals and a reduced intracellular pH) reprograms the network topology of biotic signals driving differentiation of neural progenitor cells. HDAC: Histone deacetylase, pH(i): intracellular pH.

In parallel to generation of abiotic signals, recent evidence suggests that mitochondria attenuate biotic signals during neural differentiation (Vujovic et al., 2024) (Figure 2). Upon induction of differentiation, mitochondrial outer membrane transiently fuses with the nuclear membrane followed by acquisition and degradation of nuclear-encoded RNAs in the mitochondrial intermembrane space (Vujovic et al., 2024). A further corollary of this inter-organellar communication is reprogramming of mitochondrial metabolism, suppression of ATP synthesis and switching to ATP hydrolysis by F_1_F_0_ ATP synthase. Depletion of mRNAs and a reduced energetic budget for protein synthesis combined with enhanced autophagic flux leads to attenuation of biotic signals.

## Proposal for a chimeric neural differentiation landscape: a continuum of early and late organisers

A growing body of evidence suggests that assumption of a differentiated state by neural progenitor cells is intimately linked to heightened mitochondrial activity (Ozsoy et al., 2021; Iwata et al., 2020; Beckervordersandforth et al., 2017; Khacho et al., 2016; Khacho and Slack, 2018). Herein it is suggested that neural differentiation encompasses a chimeric landscape (Figure 2). Early commitment to differentiation, which is the subject of this review, is characterised by:

- A relative paucity of homeostatic mechanisms that buffer against abiotic signals.
- Mitochondrial attenuation of biotic signals.
- Amplified production of abiotic signals by mitochondria.

A transient switch from biotic to abiotic signalling is envisaged to contribute to termination of Notch signalling pathway and to resolution of signalling bottlenecks (i.e., I-FFLs) together with erasing of specific epigenetic markers thereby enabling progenitor cells to enter the irreversible phase of differentiation. From an experimental perspective, several methodological considerations need to be observed to study the “abiotic phase” of neural differentiation. In general, the rate of neural differentiation is measured in days if not weeks, and dynamics of differentiation are studied at correspondingly long intervals. However, emerging evidence shows that within minutes of induction of differentiation, cytosolic and nuclear dynamics are significantly modified (Rezaei-Lotfi et al., 2021; Vujovic et al., 2024). Further, experimental evidence suggests that mitochondria adapt to external temperature (Terzioglu et al., 2023). Hence, the impact of mitochondria-extrinsic heat flux must be studied shortly after induction of hyperthermia and prior to deployment of adaptive mechanisms.

## Conclusion and future directions

In conclusion, it is suggested that early stage of neural differentiation is characterised by abundance of abiotic signals of mitochondrial origin and a concomitant depletion of biotic signals. The abiotic signals, including thermal flux, redox signals (e.g., reactive oxygen spices), and a reduction of cytoplasmic/nuclear pH, are triggered by mitochondrial hyperactivity, and induce a late stage of differentiation by reprogramming the network topology of biotic signals. In this process, paradoxical (i.e., antagonistic) network elements that function as signal buffering mechanisms are transiently rewired by abiotic signals to drive the progenitor cells towards a tipping point of differentiation. Exploring this proposal requires development of tools and methodologies that enable studying the rapid molecular dynamics of early differentiation cascade. Considering the short half-life of thermal and redox signals discussed within the text, the reprogramming is likely to occur at sub-minute intervals. Further, precise measurement and manipulation of abiotic signals requires development of novel tools.
